# Evaluation of Non-Structural Protein-1(NS1) positive patients of 2013 dengue outbreak in Khyber Pakhtunkhwa, Pakistan

**DOI:** 10.12669/pjms.331.11237

**Published:** 2017

**Authors:** Ghosia Lutfullah, Jawad Ahmed, Aftab Khan, Hina Ihsan, Jamshed Ahmad

**Affiliations:** 1 Dr. Ghosia Lutfullah, PhD Chemistry. Centre of Biotechnology and Microbiology, University of Peshawar, Peshawar, Pakistan; 2 Dr. Jawad Ahmad, PhD Chemistry. Department of Microbiology, Khyber Medical University Peshawar, Peshawar, Pakistan; 3 Mr. Aftab Khan, M. Phil Microbiology. PMRC Research Centre, Khyber Medical College Peshawar, Peshawar, Pakistan; 4 Miss Hina Ihsan, M. Phil. Centre of Biotechnology and Microbiology, University of Peshawar, Peshawar, Pakistan; 5 Dr. Jamshed Ahmad, PhD Microbiology. Centre of Biotechnology and Microbiology, University of Peshawar, Peshawar, Pakistan

**Keywords:** Dengue infection, Dengue diagnosis, NS1 positive samples, ELISA

## Abstract

**Background & Objective:**

Dengue infection is an arthropod borne disease caused by Dengue virus in humans. Dengue virus infection has more potential to produce severe form of the disease with more severe symptoms. Proper diagnosis of dengue fever is very important for its safe management. The objective of this study was to evaluate the non structural protein-1 (NS1) positive parameter for identification of dengue fever by using ELISA from 2013 dengue outbreak in Khyber Pakhtunkhwa.

**Methods:**

It was a cross sectional study conducted among 384 patients tested for dengue admitted to different hospitals of Khyber Pakhtunkhwa April to December 2013 with symptoms related to classical dengue fever. Written informed consent was taken from 100 NS1 positive diagnosed patients, and 3 to 5 ml blood sample was collected for confirmation through ELISA testing. ELISA test for dengue IgG and IgM was performed two time in order to confirm the dengue cases. Data was entered and analyzed by using SPSS version 16.

**Result:**

The study performed on 100 NS1 positive samples of patients, admitted to hospitals with symptoms related to classical dengue fever, indicated that after performing the IgM and IgG capture ELISA test only 76 samples were actually found positive for dengue. The rest of the 24 samples were found negative for both IgM and IgG capture ELISAs. The study also revealed that 90.8 % patients had primary dengue infection and 35.5% patients had secondary dengue infection. Most patients were between the age of 10-20 years (26%), among them19.7% were having primary dengue infection. Among 10-20 years of age 50% female patients were false dengue patients.

**Conclusion:**

About 24 % NSI protein positive samples were found negative for both IgM and IgG capture ELISAs showed that NS1protein positivity does not confirm actual dengue infection.

## INTRODUCTION

Dengue virus (DENV) is a causative agent of Dengue fever. Dengue virus (DENV) has four antigenically distinct serotypes (DENV-1, DENV-2, DENV-3, and DENV-4) all of which are able to cause complete infection.^1^ Dengue virus is an RNA virus that belongs to the family Flaviviridae, genus Flavivirus. Other viruses belonging to the same genus include Yellow fever virus, Japanese encephalitis virus, St Louis encephalitis virus that are also prevalent in Pakistan and which antigenically cross react with Dengue virus.^2^ Aedes mosquito, mainly female Aedes aegypti and Aedes albopictus serve as the transport vehicle for the dengue virus into humans.^3^

Like all other viruses Dengue virus is also not visible to the naked eye and can be seen only under electron microscope. So Dengue virus is a small virus whose genome is comprised of single strand of positive sense RNA (ssRNA) that is approximately 11kb in length. This ssRNA genome encodes merely ten proteins. Out of these ten proteins three are the structural proteins and the rest seven are known as non-structural proteins. The three structural proteins are: E protein (Envelope protein), M protein (Membrane protein), C protein (Capsid protein) and non structural proteins. The seven non-structural proteins are: NS1, NS2a, NS2b. NS3, NS4a, NS4b and NS5.^4^

The infection caused by dengue virus is known as Dengue infection or Dengue fever (DF). Symptoms of classical dengue fever include sudden high grade fever, headache, retro bulbar pain, severe pain in muscles and joints, nausea, vomiting and skin rash.^5^ However in a small number of cases the disease complications increase and it develops into a fatal dengue haemorrhagic fever (DHF) and dengue shock syndrome (DSS). Dengue haemorrhagic fever is an unusual complication of dengue infection whose symptoms include high grade fever, damage to lymph and blood vessels, thrombocytopenia and leakage of blood plasma, bleeding from gums and nose, liver enlargement and circulatory system failure. Dengue shock syndrome is another uncommon complication of dengue infection including symptoms of massive bleeding, severe leakage of plasma, nearly undetectable blood pressure and multiple organ failure.^6^

Dengue has become a global health problem infecting millions of individuals annually from the last few decades.^7^ Since its first report in 1994 from the metropolitan city of Karachi dengue virus infection has become one of the expected causes of morbidity and mortality in Pakistan.^8^,^9^ A study was conducted at Jinnah hospital Lahore Pakistan from August to November 2012 during dengue outbreak, where dengue IgM testing was done through ELISA and did not rely on NS1 positivity.^10^ The dengue NS1 antigen based devices are least efficient for accurate testing of dengue cases, because we could not differentiate between primary and secondary infection.^11^ In Pakistan, most of the clinical diagnostic facilities are using immunochromatographic (ICT) rapid tests completely relying on the supporting literature of efficacy. No local data on the sensitivity and efficacy of these kits are documented.^12^

A number of tests are currently being used for diagnosis of dengue infection which include detection of virus through cell culture, detection through RT-PCR, NS1 based assays, Immunohistochemistry, IgM based-assays and IgG based-assays but all of them have their own pros and cons and no single test gives 100% specificity and sensitivity.^13^

In Pakistan diagnosis of dengue in majority of hospitals are carried out by NS1 test, Patients are put on dengue therapy once there is NS1 positive result. Our objective was the evaluation of NS1 protein positivity for accurate diagnosis of dengue fever which was confirmed by ELISA testing for dengue IgM and IgG antibody.

## METHODS

It was descriptive cross sectional study conducted among patients admitted in different hospitals of Khyberpakhtunkhwa with symptoms related to classical dengue fever during the dengue outbreak from the duration of April to December 2013. A total 385 patients were admitted, and 100 patients were tested NS1 positive who were selected for the present study. Written informed consent was taken from these patients, and 3 to 5 ml blood sample was collected for dengue IgG and IgM antibody testing through ELISA. Age range of the study population was 1-70 years. The admitted patients were with a defined clinical symptoms and history of acute febrile illness of atleast one week with or without hemorrhage. In the present study any admitted patient tested positive for NS1 protein through ICT method in the diagnostic facility of the respective hospital was selected for further testing of dengue IgG and IgM.

### Dengue Antibodies ELISA

Dengue IgG captured ELISA (IgG-Cap-ELISA) and IgM captured ELISA (IgM-Cap-ELISA) were used to detect IgG and IgM antibodies for confirmation and differentiation of secondary and primary infection as per manufacturer instruction. 100 µl/well patient sera (diluted 1:100) and controls were added to the human IgG and human IgM coated assay plates separately. Both plates were incubated for one hour at 37 °C. Then the plates were washed and 100 μl/well of conjugate enzyme was transferred except the blank wells. After 20 minutes the reaction was stopped by adding 100 μl/well of 1 M sulphuric acid. The OD values were determined at 450nm. The automated program in ELISA machine according to the kit manufacturer protocol provided positive and negative results.

### Statistical Analysis

The quantitative variables were expressed as mean, standard deviation, minimum and maximum range, while qualitative variables were mentioned as percentages. SPSS version 16 were used for statistical analysis of the data.

## RESULTS

Among100 NS1 positive samples collected 69 were males 31 were females (Table-I). After assaying 100 samples through ELISA according to the kit manufacturer’s protocol, 76 samples were found positive for classical dengue fever and 24 samples were found negative (Table-II).

**Table-I:** Age and gender wise distribution of NS1 positive dengue patients: N= 100.

No	Age range	Male	Female	Total
1	1-10Years	9	3	12
2	11-20 years	18	6	24
3	21-50 years	32	18	50
4	50-70years	10	4	14

	Total	69	31	100

**Table-II:** Dengue Fever in NSI positive patients.

No	Age range (Years)	Positive Dengue fever cases (DF)			False dengue patient (N-DF)		

		Male No	Female No	Total	Male No	Female No	Total
1	1-10	7	2	9	2	1	3
2	11-20	15	3	18	3	3	6
3	21-50	23	15	38	9	3	12
4	51-70	9	2	11	3	0	3

	Total	54	22	76	18	6	24

The samples were assayed for the detection of both IgM and IgG antibodies against antigen of dengue virus separately. It showed that 90.8% samples ware positive for IgM and 35.5% patients have secondary dengue infection i.e positive IgG antibody (Table III and IV). The most patients were of age range 10-20 years (26%), among them19.7% were having primary dengue infection. Among 10-20 years of age 50% female patients were false dengue patients.

**Table-III:** Pattern of IgM antibody in NS1 Positive patients.

No	Age range (Years)	Positive IgM Ab			Negative IgM Ab		

		Male No (%)	Female No (%)	Total	Male No (%)	Female No (%)	Total
1	1-10	7 (9.2)	2 (2.6)	9 (11.8)	1 (1.3)	0 (0)	1 (1.3)
2	11-20	15 (19.7)	3 (4)	18 (23.7)	2 (2.6)	0 (0)	2 (2.6)
3	21-50	20 (26.3)	12 (15.8)	32 (42)	3 (2.6)	1 (1.3)	4 (5.2)
4	51-70	8 (10.5)	2 (2.6)	10 (13)	0 (0)	0 (0)	0 (0)

	Total	50 (65.8)	19 (25)	69 (90.8)	6 (7.9)	1 (1.3)	7 (9.2)

**Table-IV:** Pattern of IgG antibody in NS1 Positive patients.

No	Age range (Years)	Positive IgG Ab			Negative IgG Ab		

		Male No (%)	Female No (%)	Total	Male No (%)	Female No (%)	Total
1	1-10	2 (2.6)	0 (0)	2 (2.6)	5 (6.5)	1 (1.3)	6 (7.9)
2	11-20	6 (7.9)	1 (1.3)	7 (9.2)	9 (11.8)	2 (2.6)	11(14.4)
3	21-50	11 (14.4)	5 (6.5)	16 (21)	19 (25)	6 (7.9)	25 (32.9)
4	51-70	2 (2.6)	0 (0)	2 (2.6)	5 (6.5)	2 (2.6)	7 (9.2)

	Total	21 (27.6)	6 (7.9)	27(35.5%)	38 (50)	11 (14.4)	49

## DISCUSSION

A number of rapid dengue diagnostic tests are available commercially and several in-house assays have also been developed but the performance of these tests has not been evaluated adequately. In an international meeting held at World Health Organization in Geneva on dengue diagnosis in October 2004 and April 2005, a need for the development of an authenticated diagnostic test for the diagnosis of dengue infection was suggested.^13^ There is a need for a sensitive, specific and less expensive diagnostic test for dengue virus detection which can be utilized for surveillance, clinical management and for investigations of outbreak which would allow early treatment of patients and control or prevent epidemics.

The antigen non-structural protein-1 is highly immunogenic and has a relationship to dengue viremia. Many assays including immunochromatographic tests and enzymatic immunoassays can detect NS1 in serum, plasma and whole blood. Routine diagnosis of dengue is carried out by this method worldwide as the performance was evidenced to be good. However, different groups from several countries have showed that NS1 assays have low sensitivity comparatively, specifically in the populations experiencing several dengue outbreaks. A multi-country study was carried out in 6 countries of the Americas and Asia. This study suggested that the NS1 assay which gives the best performance showed intermediate sensitivity (median 64%, range 34% to 76%) and high specificity for dengue diagnosis. When in the samples the detection of NS1 and IgM was combined it increased the overall sensitivity of dengue diagnosis.^14^

A huge outbreak occurred in Santos, Brazil in 2010. For the diagnosis of dengue NS1 antigen detection was carried out along with the detection of IgM, IgG and RNA. False NS1 negative results were obtained in a large number. For evaluation 379 samples were chosen which were RNA-positive. Only in 37.7% cases NS1 was found to be reactive. Several samples were characterized as secondary dengue infections by DENV-2. The isolates of NS1 positive and negative were sequenced which did not show any kind of mutation which could give good reason for why the diagnosis failed.^15^

In another study six assays were performed on the samples of 259 patients from Sri Lanka who had acute fevers in which 99 were confirmed for dengue and 160 patients were confirmed for other acute febrile illnesses. The detection of NS1 antigen showed sensitivity from 49% to 59% and detection of IgM antibodies showed sensitivity from 71% to 80%. By combining the results of IgM antibody and NS1 antigen best sensitivity of 93% was observed.^16^

The sensitivity of NS1 ELISA assay was evaluated by Hang et al. using samples of Vietnamese adults and children and the result was compared with a standard reference diagnostic algorithm. Two types of NS1 test were used for diagnosis which suggested the higher sensitivity of the test when it is carried out in the first three days of infection and which are more likely to be positive if the patient is suffering from primary dengue infection. The study also suggested that a positive NS1 test has a relationship with the concentration of virus in the blood. So those patients who have greater concentration of virus are more likely to be positive for NS1 test. So collectively the study pointed out that NS1 tests require some inclusions in its diagnostic approach towards dengue.^17^

The evaluation of two enzyme linked immunosorbant assays for the diagnosis of dengue infection was carried out by Blacksell et al. One was a test performed for the detection of immunoglobulin M antibody and another test was based on dengue virus non-structural protein 1 detection. About 41% of the patients were confirmed for dengue in which 11% had acute primary and 87% had secondary infections. The NS1 test sensitivity was 63% on admission samples and on convalescent samples the sensitivity further decreased. While the IgM capture ELISA showed higher sensitivity on convalescent samples as compared to the admission samples. Then NS1 and IgM capture ELISA results were combined which increased the sensitivity for both the admission and convalescent samples.^18^

Present study is in accordance with the said reports as the sensitivity of NS1 test was less towards the diagnosis of dengue because after passing the samples through assays of IgM and IgG capture ELISAs separately 24% of the result was found negative.

One of the limitations of our study is the actual clarity of NS1 protein detection methods in hospitals. Mostly we were using ICT strips and few hospitals did not clarify it.

## CONCLUSION

Study showed that 24% NSI protein positive samples were found negative for both IgM and IgG capture ELISA showed that NS1 protein positivity does not confirm actual dengue infection. Dengue infection must be confirmed through more sensitive tests. Dengue infection was high in age group 10-20 years particularly among male genders, need counseling and awareness program for the youth to follow the precaution rules of dengue infection and to discourage the outdoor activities of youth during mosquito biting time. High rate of false dengue infection in females of age range 10-20 shows the low importance of NS1 protein in diagnosis and need of more sensitive test for proper diagnosis.

## References

[1] Normile D (2013). Surprising new dengue virus throws a spanner in disease control efforts. Science.

[2] Gould E, Solomon T (2008). Pathogenic flaviviruses. Lancet.

[3] Das S, Pingle MR, Munoz-Jordan J, Rundell MS, Rondini S, Granger K (2008). Detection and serotyping of dengue virus in serum samples bymultiplex reverse transcriptase PCR-ligase detection reaction assay. J Clin Microbiol.

[4] Perera R, Kuhn RJ (2008). Structural proteomics of dengue virus. Curr Opin Microbiol.

[5] Vaughn DW, Green S, Kalayanarooj S, Innis BL, Nimmannitya S, Suntayakorn S (2000). Dengue viremia titer, antibody response pattern, and virus serotype correlate with disease severity. J Infect Dis.

[6] Organization WH, Research SPf Diseases TiT Diseases WHODoCoNT Epidemic WHO Alert P (2009). Dengue: guidelines for diagnosis, treatment, prevention and control. World Health Organization.

[7] Gubler DG (2002). The global emergence/resurgence of arboviral diseases as public health problems. Arch Med Res.

[8] Chan YC, Salahuddin NI, Khan J, Tan HC, Seah CL (1995). Dengue haemorrhagic fever outbreak in Karachi, Pakistan 1994. Trans R Soc Trop Med Hyg.

[9] Wasay M, Channa R, Jumani M, Zafar A (2008). Changing patterns and outcome of dengue infection;report from a tertiary care hospital in Pakistan. J Pak Med Assoc.

[10] Assir MZ, Masood MA, Ahmad HI (2014). Concurrent dengue and malaria infection in Lahore, Pakistan during the 2012 dengue outbreak. Int J Infect Dis.

[11] Naz A, Zahid D, Mukry SN, Nadeem M, Sil BK, Shamsi TS (2014). Evaluation of efficacy of various immunochromatographic rapid tests for dengue diagnosis. Pak J Med Sci.

[12] Hakim ST, Tayyab SM, Qasmi SU, Nadeem SG (2011). An experience with dengue in Pakistan: An expanding problem. Ibnosina J Med BS.

[13] Peeling RW, Artsob H, Pelegrino JL, Buchy P, Cardosa MJ, Devi S (2010). Evaluation of diagnostic tests: dengue. Nature Rev Microbiol.

[14] Guzman MG, Jaenisch T, Gaczkowski R, Ty Hang VT, Sekaran SD, Kroeger A (2010). Multi-country evaluation of the sensitivity and specificity of two commercially-available NS1 ELISA assays for dengue diagnosis. PLoS Negl Trop Dis.

[15] Felix AC, Romano CM, de Campos Centrone C, Rodrigues CL, Villas-Boas L, Araújo ES (2012). Low sensitivity of NS1 protein tests evidenced during a dengue type 2 virus outbreak in Santos, Brazil, in 2010. Clin Vaccine Immunol.

[16] Blacksell SD, Jarman RG, Bailey MS, Tanganuchitcharnchai A, Jenjaroen K, Gibbons RV (2011). Evaluation of six commercial point-of-care tests for diagnosis of acute dengue infections: the need for combining NS1 antigen and IgM/IgG antibody detection to achieve acceptable levels of accuracy. Clin Vaccine Immunol.

[17] Hang VT, Nguyet NM, Tricou V, Yoksan S, Dung NM, Van Ngoc T (2009). Diagnostic accuracy of NS1 ELISA and lateral flow rapid tests for dengue sensitivity, specificity and relationship to viraemia and antibody responses. PLoS Negl Trop Dis.

[18] Blacksell SD, Mammen MP, Thongpaseuth S, Gibbons RV, Jarman RG, Jenjaroen K (2008). Evaluation of the Panbio dengue virus nonstructural 1 antigen detection and immunoglobulin M antibody enzyme-linked immunosorbentassays for the diagnosis of acute dengue infections in Laos. Diagn Microbiol Infect Dis.

